# Supplementary material for: Content-rich biological network constructed by mining PubMed abstracts
Source: BMC Bioinformatics. 2004 Oct 8;5:147. doi: 10.1186/1471-2105-5-147 (PMC528731; doi:10.1186/1471-2105-5-147)
Supplement: Additional File 2 — The original results of the above study (non-essential files are deleted to keep the file size under the limit set by BMC bioinformatics). [file 1471-2105-5-147-S2.bz2 › chilibotAdditionalFile2/dip10/680ID7658473E11090/html/CTXA_CTXB.html]

 


 **CTXA** and **CTXB** 
  
Found 12 abstracts in PubMed, retrieved 10.  
 

 What does Google say? 
 PDF only 
| .edu only 

---

**Interactive relationship** (e.g. stimulation, inhibition, etc)

**Non-interactive relationship** (e.g. studied together, co-existance, homology, etc.)

- it controls expression of toxin genes  **ctxA**  and  **ctxB** , along with a variety of other genes related to pathogenicity.  Ref: 7664730 EMBO J, 1995
- Among these were three V. cholerae strains that contained CTX prophage sequences but not the  **ctxA**  and  **ctxB**  genes.  Ref: 10986258 J Bacteriol, 2000
- The genes encoding cholera toxin  **ctxA**  and  **ctxB**  are encoded in the genome of CTXphi, a filamentous phage that infects Vibrio cholerae.  Ref: 10986258 J Bacteriol, 2000
- PCR analysis showed that 71% of O1 clinical isolates had a complete set of CTX element target genes  **ctxA** ,  **ctxB** , zot and ace and 68% a complete set of the VPI genes studied orf1, aldA, tagA, tcpA, toxT and int genes.  Ref: 12393195 FEMS Microbiol Lett, 2002
- Comparison of cholera toxin CT production directed by different gene constructs and S1 nuclease mapping revealed the presence of a  **ctxB**  specific promoter within the  **ctxA**  coding sequence.  Ref: 9119505 Infect Immun, 1997
- PCR amplification with primers specific to the  **ctxA** ,  **ctxB** , tcpA, tagA, int, aldA, toxT, LJ, RJ and toxR genes revealed that six of the seven rough isolates were positive for these genes.  Ref: 11232774 J Med Microbiol, 2001
- V, cholerae strains carrying  **ctxA** \*, with either  **ctxB**  1 or  **ctxB**  2, and inactivated zot genes were created by homologous recombination.  Ref: 8039872 Infect Immun, 1994
- A concentrated protein sample from the 37 degrees C overnight culture supernatant of CVD110, a delta  **ctxA** , delta zot, delta Ace and hlyA  **ctxB**  mer mutant of El Tor biotype Ogawa serotype strain E7946 caused morphological changes in cultured MDCK I epithelial cells and altered their arrangement of filamentous actin F actin and Zonula occludens associated protein ZO 1.  Ref: 8844654 Microb Pathog, 1996
- sequence data were also obtained for the virulence associated genes tcpA,  **ctxA** , and  **ctxB** .  Ref: 12734277 J Clin Microbiol, 2003
- Restriction fragment length polymorphisms of rRNA and  **ctxA**  genes, DNA sequence of cholera toxin B subunit gene  **ctxB** , and multilocus enzyme electrophoresis data were used to characterize 197 isolates.  Ref: 7680060 J Infect Dis, 1993
- When A further deletion from XbaI to ClaI of cholera toxin A gene about 550bp was made and  **ctxA**  was outframe with LacZ,  **ctxB**  expression was decreased two fold compared to pUC19CTB.  Ref: 7718277 Yi Chuan Xue Bao, 1994
- The results also showed that 72.4% of environmental O1 isolates possessed  **ctxA** ,  **ctxB** , zot and ace genes while environmental non O1 strains rarely possessed virulence genes.  Ref: 12393195 FEMS Microbiol Lett, 2002
